# Stabilization of SAMHD1 by NONO is crucial for Ara-C resistance in AML

**DOI:** 10.1038/s41419-022-05023-0

**Published:** 2022-07-08

**Authors:** Feifei Zhang, Jun Sun, Xiaofeng Tang, Yiping Liang, Quanhui Jiao, Bo Yu, Zhengzai Dai, Xuhui Yuan, Jiayu Li, Jinhua Yan, Zhiping Zhang, Song Fan, Min Wang, Haiyan Hu, Changhua Zhang, Xiao-Bin Lv

**Affiliations:** 1 Jiangxi Key Laboratory of Cancer Metastasis and Precision Treatment, Central Laboratory, The First Hospital of Nanchang, The Third Affiliated Hospital of Nanchang University, North 128 Xiangshan Road, Nanchang, 330008 China; 2 College of Pharmacy, Jiangxi University of Chinese Medicine, Nanchang, 330004 China; 3 Department of Orthopedics, The First Hospital of Nanchang, The Third Affiliated Hospital of Nanchang University, North 128 Xiangshan Road, Nanchang, 330008 China; 4 Department of Oral and Maxillofacial Surgery, Sun Yat-Sen Memorial Hospital of Sun Yat-Sen University, Guangzhou, 510120 China; 5 Tianjin Key Laboratory of Lung Cancer Metastasis and Tumor Microenvironment, Tianjin Lung Cancer Institute, Tianjin Medical University General Hospital, Tianjin, 300052 China; 6 Oncology Department of Shanghai Jiao Tong University Affiliated Sixth People’s Hospital, Shanghai, 200233 China

**Keywords:** Drug development, Ubiquitylation, Oncogenes

## Abstract

Cytarabine (Ara-C) is the first-line drug for the treatment of acute myelogenous leukemia (AML). However, resistance eventually develops, decreasing the efficacy of Ara-C in AML patients. The expression of SAMHD1, a deoxynucleoside triphosphate (dNTP) triphosphohydrolase, has been reported to be elevated in Ara-C-resistant AML patients and to play a crucial role in mediating Ara-C resistance in AML. However, the mechanism by which SAMHD1 is upregulated in resistant AML remains unknown. In this study, NONO interacted with and stabilized SAMHD1 by inhibiting DCAF1-mediated ubiquitination/degradation of SAMHD1. Overexpression of NONO increased SAMHD1 expression and reduced the sensitivity of AML cells to Ara-C, and downregulation of NONO had the opposite effects. In addition, the DNA-damaging agents DDP and adriamycin (ADM) reduced NONO/SAMHD1 expression and sensitized AML cells to Ara-C. More importantly, NONO was upregulated in Ara-C-resistant AML cells, resulting in increased SAMHD1 expression in resistant AML cells, and DDP and ADM treatment resensitized resistant AML cells to Ara-C. This study revealed the mechanism by which SAMHD1 is upregulated in Ara-C-resistant AML cells and provided novel therapeutic strategies for Ara-C-resistant AML.

## Introduction

Acute myelogenous leukemia (AML) is a hematological malignancy arising from abnormal proliferation of myeloid leukocytes, with overall five-year survival rates of approximately 10% in elderly individuals and 70% in children [1]. As one of the main drugs for the treatment of AML, cytarabine (Ara-C) is usually used clinically in combination with anthracyclines to achieve the best therapeutic effect [2, 3]. Ara-C enters the cell and is converted into a therapeutically active triphosphate metabolite, Ara-CTP, which enters the nucleus and inhibits DNA synthesis, in turn triggering apoptosis and exerting antileukemic effects [4–6]. However, resistance to Ara-C eventually develops in AML patients, and the emergence of resistance poses a great therapeutic challenge [7, 8]. Sterile alpha motif (SAM) and histidine-aspartate (HD) domain-containing protein 1 (SAMHD1) have been reported to negatively regulate the level of Ara-CTP, leading to a decrease in its intracellular level, and SAMHD1 is highly upregulated in Ara-C-resistant AML patients, thus decreasing their sensitivity to Ara-C [9, 10]. Therefore, SAMHD1 has been recognized as a crucial contributor to Ara-C resistance in AML patients and an important therapeutic target for Ara-C-resistant AML patients.

SAMHD1, an adenosine triphosphate hydrolase, plays an important role in cancer development, antiviral immune responses, and DNA damage repair and chemoresistance [11–15]. SAMHD1 plays dual roles in cancers. On the one hand, SAMHD1 plays tumor-suppressive roles in a variety of cancers. For instance, SAMHD1 mediates the phosphorylation of p27^kip1^ through regulation of the PI3K-Akt signaling pathway and subsequently affects the proliferation of AML cells [11] and inhibits lung adenocarcinoma progression through negative regulation of STING [16]. On the other hand, SAMHD1 has been implicated in the chemotherapeutic resistance of AML cells to nucleoside antimetabolites. Knockout of SAMHD1 was linked to increased sensitivity to the antimetabolites nelarabine, fludarabine, decitabine, vidarabine, clofarabine, and trifluridine [17, 18].

SAMHD1 is regulated both transcriptionally and posttranscriptionally. SAMHD1 is transcriptionally downregulated by promoter hypermethylation in lung cancer cells and in CD4 T cells during viral infection [19, 20]. Phosphorylation at threonine 592 (T592) by cellular cyclin-dependent kinases abrogates SAMHD1 activity, which reduces its antiviral ability or interferes with cellular DNA replication and S phase progression [21]. Acetylation of SAMHD1 at lysine 405 (K405) increases deoxynucleoside triphosphatase (dNTPase) activity and promotes cancer cell proliferation [22]. In addition, SUMOylation of SAMHD1 at lysine 595 has been reported to increase antiviral activity in noncycling immune cells [23]. The stability of SAMHD1 is also regulated by posttranslational modification. For example, Li et al. [24] reported that the TRIM21 E3 ligase ubiquitinates and degrades SAMHD1 in rhabdomyosarcoma RD cells upon enterovirus 71 (EV71) infection. In addition, the virus-derived protein VPX can hijack the Cul4-DDB1-DCAF1 E3 ligase complex and mediate the degradation of SAMHD1, facilitating viral infection [25]. However, the mechanism by which SAMHD1 is upregulated in resistant AML remains unknown.

In this study, we report that NONO interacts with and stabilizes SAMHD1 by inhibiting its ubiquitination/degradation mediated by the DDB1-DCAF1 E3 ligase. The upregulation of NONO in resistant AML cells leads to an increased level of SAMHD1 and contributes to the resistance of AML cells to Ara-C. Suppression of NONO expression using siRNA or chemical agents partially restores the sensitivity of resistant AML cells to Ara-C. Our findings reveal the mechanism of SAMHD1 upregulation in resistant AML cells and provide a potential strategy to overcome Ara-C resistance in AML.

## Materials and methods

### Western blotting and coimmunoprecipitation (co-IP)

Cells were lysed with RIPA buffer (50 mM Tris–HCl, 150 mM NaCl, 5 mM EDTA, and 0.5% Nonidet P-40) supplied with a protease inhibitor cocktail (Millipore, 539131-1VL) on ice for 30 min and were then centrifuged at 14,000 rpm for 30 min at 4 °C. Supernatants were added to a 1/5 volume of 5× SDS loading buffer, boiled for 10 min, separated by SDS–PAGE, and transferred to polyvinylidene fluoride (PVDF) membranes.

The membranes were incubated with primary antibodies overnight at 4 °C and with secondary antibodies for one hour at room temperature. After extensive washing, the membranes were visualized with an ECL kit (Beyotime Ltd., Shanghai, China) for protein detection. Antibodies against the Myc tag (3946 S), HA tag (3724 S), and Flag tag (2368 S) were purchased from Cell Signaling Technology (Boston, USA). The antibodies against NONO (11058-1-AP), SAMHD1 (12586-1-AP), DCAF1 (11612-1-AP), and Actin (20536-1-AP) used for western blotting were purchased from Proteintech (Wuhan, China). The antibodies against NONO (ab70335) and SAMHD1 (ab264335) used for co-IP were obtained from Abcam (Cambridge, UK).

### Reverse transcription (RT) and real-time PCR

Total RNA was extracted from cultured cells using TRIzol reagent (Life, USA) according to the manufacturer’s instructions and reverse transcribed into cDNA using a PrimeScript™ RT Reagent Kit with gDNA Eraser (Takara, Japan). Quantitative analysis was performed using RealMaster Mix (SYBR Green Kit, Takara, Shiga, Japan) on a CFX96 Real-Time PCR Detection System (Bio–Rad Laboratories, RRID: SCR_008426). RNA levels were calculated and normalized to GAPDH. The primers used for quantitative RT–PCR (qRT–PCR) are listed in Supplementary Table S.

### Cell viability assay

Parental and Ara-C-resistant AML cells were cultured overnight in 24-well plates and treated with the indicated concentrations of drugs for 48 h. CCK-8 reagent was then added to the culture medium and incubated for 1 h. The supernatant was collected and added to a 96-well plate, and the OD value was measured at 450 nm. The drug combination assay was performed according to Chou–Talalay method, and the combination index (CI) used to assess the synergistic effects was calculated with CalcuSyn software (Biosoft). The CI quantitatively defines the synergistic effect of a drug combination as follows: CI = 1 indicates an additive effect, CI < 1 indicates synergism, and CI > 1 indicates antagonism.

### Apoptosis assay

The apoptosis assay was performed using AnnexinV-PI apoptosis detecting kits (KGA108, KeyGEN BioTECH, China) according to the manufactory’s instructions. Briefly, around 2 × 10^5^ AML cells for each test were collected and washed twice with PBS. Then the cells suspended with 500 μL binding buffer were stained with Annexin V-FITC and propidium iodide (PI) solutions for 15 min in the dark. The apoptosis rate was detected using flow cytometers (BD Biosciences).

### Cell culture and reagents

HL60, THP-1, MV4-11, HEL, K562, and 293 T cells were obtained from Shanghai Institute of Cell Science, Chinese Academy of Sciences. All cell lines were cultured in RPMI 1640 medium (Thermo Fisher Scientific, Waltham, MA, USA) supplemented with 10% FBS and were maintained at 37 °C in 5% CO_2_. All cells were subjected to short tandem repeat profiling and an incubation period of no more than two months. Ara-C, adriamycin (ADM), DDP, paclitaxel, 5-fluorouracil (5-FU), and methotrexate (MTX) were purchased from MedChemexpress CO., Ltd. Ara-C-resistant HEL (HLE-R) and HL60 (HL60-R) cells were generated by culturing cells in medium with a gradient of progressively increasing drug concentrations, with a final concentration of 8 μM. For all assays, drug-resistant cells were cultured for 48 h in drug-free medium before use in experiments.

### Co-IP

For endogenous IP, AML cells were lysed on ice for 30 min using RIPA lysis buffer and centrifuged at 14,000 rpm for 30 min. The supernatant was incubated with 4 μg of the anti-NONO or anti-SAMHD1 antibody for 3 h at 4 °C, and IgG was used as the negative control. Then, the antibody complexes were precipitated using protein A/G agarose, washed 5 times with RIPA buffer, and boiled with 1× loading buffer for 10 min. For exogenous IP, cell lysates were incubated with anti-HA (A2095, Sigma–Aldrich, St. Louis, USA) or anti-Myc (sc-40AC, Santa Cruz, California, USA) agarose for 4 h at 4 °C or overnight. Then, the agarose was washed extensively with RIPA buffer, added to 1× SDS loading buffer, and boiled for 10 min. Proteins were then detected by western blotting.

### GST pulldown assay

The plasmids encoding GST-NONO and GST-SAMHD1 were transformed into *E. coli* BL21 cells. When the cells were grown up to OD value 0.8, the cells were added with 50 ug/mL of Isopropyl β-D-thiogalactoside (IPTG) (MedChemExpress) and cultured at 22 °C for another 6 h. Then the cells were resuspend with Tris-HCl buffer (50 mM Tris-HCl, 150 mM NaCl and 1× protein inhibitor cocktail, pH 8.0) and lysed by sonication. The GST-NONO, GST-SAMHD1, or GST (as a negative control) were immobilized with Glutathione-Sepharose beads (HY-K0211, MedChemexpress, New Jersey, USA) and washed 3 times with Tris-HCl buffer. The Glutathione-Sepharose beads coupled with GST-NONO, GST-SAMHD1, or GST proteins were incubated with the whole-cell lysates of the AML cells under gentle rotation for 3 h or overnight and washed intensively with RIPA buffer. After rinsing the beads three times with washing RIPA buffer, the proteins bound to the beads were boiled, separated using 10% SDS–PAGE, and visualized by western blotting.

### Ubiquitination assay

HA- or Myc-tagged plasmids were cotransfected with Flag-Ub into 293 T cells for 24 h, and the cells were treated with MG132 for 6 h before harvesting. The cells were lysed, and the supernatant was incubated with an anti-HA (A2095, Sigma–Aldrich, St. Louis, USA) or anti-Myc (sc-40AC, Santa Cruz, California, USA) antibody for 4 h or overnight. After washing extensively with RIPA buffer, the protein complexes were separated by SDS–PAGE, immunoblotted with an anti-Flag antibody, and detected with a chemiluminescence kit.

### SiRNAs, transfection, and establishment of stable cell lines

The siRNA sequences against NONO, SAMHD1, TRIM21, and DCAF1 were validated previously [9, 26–29] and were synthesized at GenePharma (Shanghai, China). Scrambled sequences purchased from GenePharma (Shanghai, China) were used as negative controls for the knockdown experiments. The siRNA targeting sequences are listed in Supplementary Table S. siRNA transfection was performed using Lipofectamine RNAiMAX (Invitrogen, Waltham, MA, USA) according to the manufacturer’s instructions. For plasmid transfection into 293 T cells or AML cells, polyethylenimine (PEI; MW = 25,000 Daltons) (Polyscience, Illinois, USA) or Lipofectamine 2000 (Invitrogen, Waltham, MA, USA) was used, respectively. To generate stable cell lines, 293 T cells cotransfected with pLKO.1-NONO or pLKO.1 and packaging/envelope plasmids (psPAX2/pMD2.G) were incubated at 37 °C for 24 h. The virus-containing supernatant was collected and used to transduce AML cells. After 2 weeks of screening with puromycin (0.5 μg/ml), cells were collected, and the silencing efficiency was determined by western blotting.

### Plasmid construction

S protein-binding peptide, streptavidin-binding peptide, and the HA tag were cloned into the pcDNA3.1 vector using the HindIII and Kpn1 endonucleases to construct the pSSH vector. The Myc-NONO, pSSH-NONO, NONO truncation mutant, and pLKO.1-NONO vectors were constructed previously [30]. The pSSH-SAMHD1 and pSSH-DCAF1 vectors were constructed by cloning the full-length SAMDH1 and DCAF1 sequences into the pSSH vector. The Myc-tagged SAMHD1 vector was constructed by cloning the full-length SAMHD1 sequence into Myc-pcDNA3.1. The SAMHD1 truncation vectors were constructed using a Q5® Site-Directed Mutagenesis Kit (New England Biolabs, USA). PrimeSTAR Max (Takara) was used for amplification, and the primer sequences used for amplification and shRNA sequences targeting NONO are listed in Supplementary Table S.

### Xenograft model

All animal experiments were approved by the ethics committee of The First Hospital of Nanchang. Four- to six-week-old female NOD/SCID mice were purchased from Shanghai Institutes for Biological Sciences, Chinese Academy of Sciences (Shanghai, China). After 2–3 days of adaptive feeding, each mouse was injected subcutaneously with 5 × 10^6^ cells in 200 µl of PBS (*n* = 6 in each group). When the tumor volume was ~150 mm^3^, the mice were randomly divided into groups. The mice in each group were injected intraperitoneally with Ara-C (75 mg/kg, once a day), DDP (75 mg/kg, once a day), or both. The size of the xenograft tumors was assessed once a day. After approximately 14 days of treatment, before the tumor size in the mice was 1500 mm^3^ ((length × width ^2^)/2), the mice were sacrificed by cervical dislocation under anesthesia, and xenograft tumors were harvested for subsequent analysis.

### Statistical analysis

Statistical analysis was performed, and data were plotted using GraphPad Prism 7 (GraphPad Software, Inc., San Diego, CA, USA). Comparisons between two groups were made using a two-tailed Student’s *t* test. All data are shown as the mean ± standard deviation (SD) values. P values less than 0.05 were considered significant. All cell culture experiments were performed three times independently.

## Results

### SAMHD1 interacts with NONO in AML cells

**Fig. 1:**
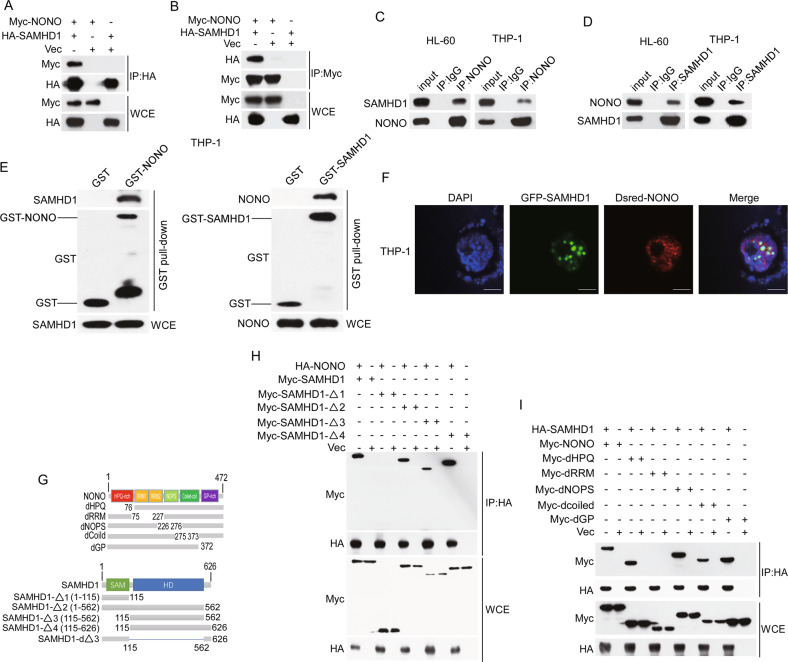
SAMHD1 interacts with NONO. **A**, **B** Validation of the exogenous interaction between NONO and SAMHD1 by co-IP. HA-tagged SAMHD1 was cotransfected with Myc-tagged NONO into 293 T cells. Co-IP was performed with anti-HA or anti-Myc agarose, and the interactions were examined by western blotting. **C**, **D** Identification of the endogenous interaction between NONO and SAMHD1 in HL60 and THP-1 cells. Cell lysates were immunoprecipitated with an anti-NONO antibody, and the indicated proteins were examined by western blotting. IgG was used as the negative control. **E** NONO interacts with SAMHD1 detected by GST pulldown assay. E. coli-expressed GST-NONO, GST-SAMHD1, or GST (as a negative control) proteins were immobilized by Glutathione-Sepharose and then incubated with THP-1cell lysate. GST or GST-NONO, GST-SAMHD1 and pull downed proteins were examined by western blotting with indicated antibodies. **F** Identification of the co-location of NONO and SAMHD1 in THP-1 cells by immunofluorescence. GFP-SAMHD1 was cotransfected with Dsred-NONO into THP-1 cells for 24 h. Scale bar, 5 μM. **G** Schematic diagram of NONO and SAMHD1 truncation mutants. **H** The domain of SAMHD1 containing amino acids 115-562 interacts with NONO. HA-tagged NONO was cotransfected into 293 T cells with wild-type or mutant Myc-SAMHD1, and the interactions were verified by co-IP and western blotting. **I** The RRM domain of NONO interacts with SAMHD1. HA-tagged SAMHD1 was cotransfected into 293 T cells with wild-type or mutant Myc-NONO, and the interactions were verified by co-IP and western blotting.

We previously found that SAMHD1 was present in the NONO complex identified by tandem affinity purification and mass spectrometry (TAP-MS) [30] (Supplementary Fig. S1A). To confirm their interaction, we transfected Myc-tagged NONO and HA-tagged SAMHD1 into 293 T cells and performed co-IP assays. As shown in Fig. 1A, B, exogenous SAMHD1 and NONO interacted with each other (Fig. 1A, B). In addition, this interaction was further confirmed by endogenous co-IP in HL60 and THP-1 cells (Fig. 1C, D). Consistently, GST pulldown assay results showed that GST-NONO, GST-SAMHD1 but not negative control GST interacted with SAMHD1 and NONO, respectively, indicating that they interacted with each other directly (Fig. 1E and Supplementary Fig. S1B). Furthermore, the immunofluorescence (IF) assay results showed that NONO and SAMHD1 were colocalized in the nucleus in THP-1 cells (Fig. 1F). To map the domains mediating the interaction of NONO and SAMHD1, we constructed a series of NONO and SAMHD1 truncation mutants (Fig. 1G). The co-IP assay results showed that the domain of SAMHD1 containing amino acids 115-562 interacted with NONO (Fig. 1H); moreover, deletion of this domain abrogated the interaction of SAMHD1 with NONO (Supplementary Fig. S1C). Moreover, deletion of the RNA recognition motif (RRM) domain of NONO abrogated its interaction with SAMHD1 (Fig. 1I), and the RRM domain alone was sufficient for the binding of NONO to SAMHD1 (Supplementary Fig. S1D).

### NONO improves the stability of SAMHD1

**Fig. 2:**
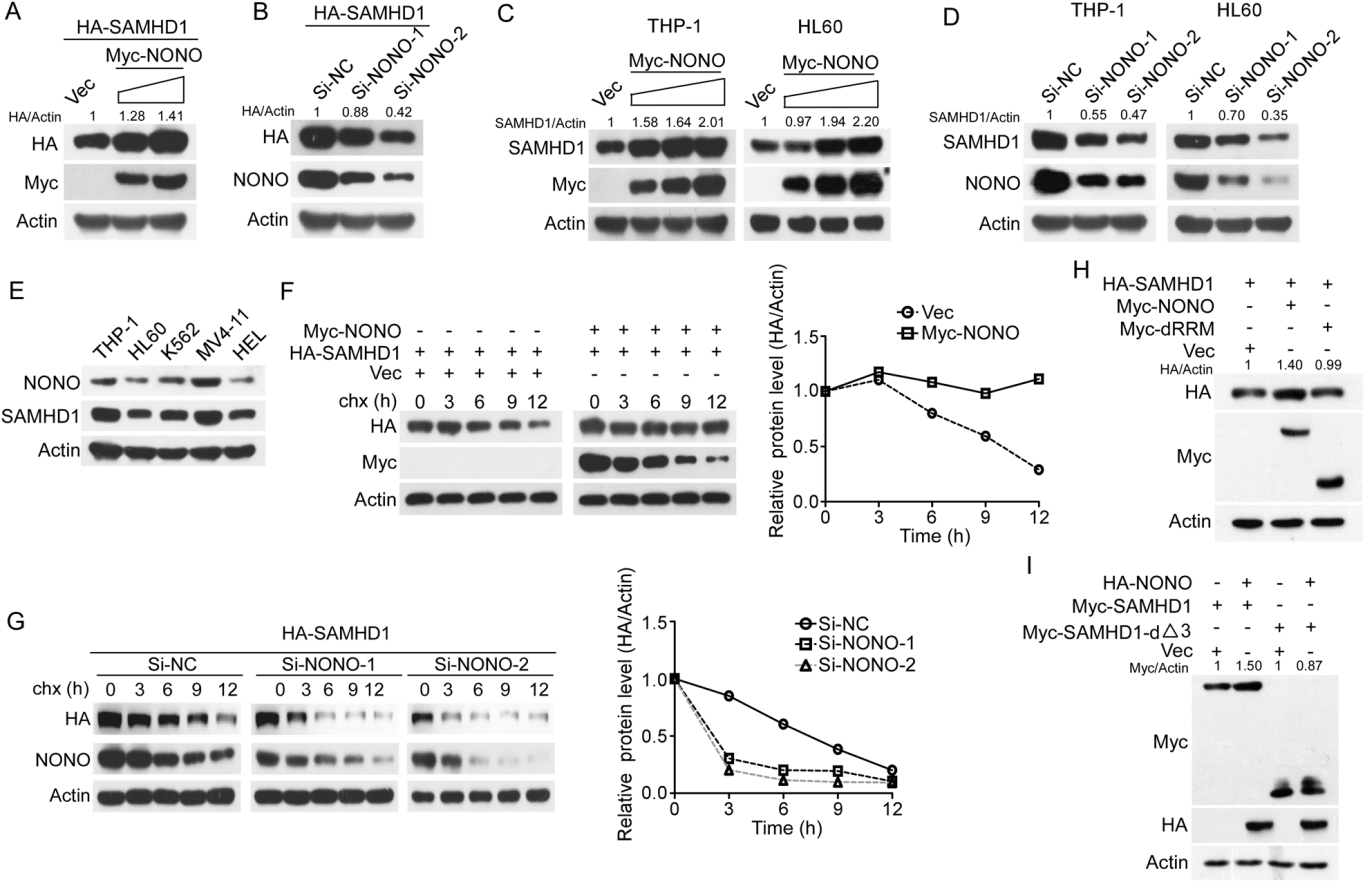
NONO improves the stability of SAMHD1. **A**–**D** Overexpression or silencing of NONO decreased or increased both the exogenous and endogenous expression of SAMHD1, respectively. **A** 293 T cells transfected with HA-SAMHD1 and increasing concentrations of Myc-NONO for 24 h were lysed and the levels of the indicated proteins were evaluated using western blotting. **B** 293 T cells transfected with NONO siRNAs for 24 h were then transfected with HA-SAMHD1 for 24 h and the levels of the indicated proteins were evaluated using western blotting. **C** AML cells were transfected with increasing concentrations of Myc-tagged NONO for 24 h, and the levels of the indicated proteins were evaluated using western blotting. **D** AML cells were transfected with NONO or negative control siRNAs for 72 h, and the levels of the indicated proteins were evaluated using western blotting. **E** Expression levels of NONO and SAMHD1 in AML lines. THP-1, HL60, K562, MV4-11, and HEL cells were cultured for 24 h, and the levels of the indicated proteins were evaluated by western blotting. **F**, **G** CHX chase assays confirmed that NONO improves the stability of SAMHD1. **F** HA-tagged SAMHD1 was cotransfected with or without Myc-tagged NONO into 293 T cells for 24 h, and the cells were treated with CHX for the indicated time before harvesting. **G** 293 T cells transfected with NONO or negative control siRNAs for 48 h were treated with CHX for the indicated time before harvesting. The levels of the indicated proteins were determined by western blotting (left) and quantitative analysis (right). **H** Deletion of the RRM domain of NONO does not increase SAMHD1 expression. HA-tagged SAMHD1 was cotransfected with wild-type or mutant Myc-NONO into 293 T cells for 24 h. The levels of the indicated proteins were determined by western blotting. **I** NONO does not affect the protein level of the SAMHD1 truncation mutant with deletion of amino acids 115 to 562. HA-tagged NONO was cotransfected with wild-type or mutant Myc-SAMHD1 into 293 T cells for 24 h. The levels of the indicated proteins were determined by western blotting. For respective immunoblots, the protein levels were quantified by ImageJ software.

Considering the interaction of NONO and SAMHD1, we sought to explore their functional interplay. First, overexpression or silencing of NONO increased or reduced the protein level of exogenous SAMHD1, respectively, in 293 T cells (Fig. 2A, B), while modulation of the SAMHD1 level had little influence on the NONO protein level (Supplementary Fig. S2A, B), indicating that NONO may affect SAMHD1 expression. In addition, the upregulation and downregulation of SAMHD1 by NONO at the endogenous protein level but not the mRNA level was confirmed in THP-1 and HL60 cells (Fig. 2C, D and Supplementary Fig. S2C, D). Furthermore, the protein level of NONO was found to be positively correlated with that of SAMHD1 in AML cells (Fig. 2E). Collectively, these results indicate that NONO regulates SAMHD1 expression at the posttranslational level. Indeed, overexpression of NONO prolonged the half-life of SAMHD1, whereas silencing of NONO shortened its half-life (Fig. 2F, G). Finally, deletion of the RRM domain of NONO or amino acids 115-562 of SAMHD1 abrogated NONO-mediated upregulation of SAMHD1 (Fig. 2H, I). Taken together, these results indicated that NONO upregulates SAMHD1 expression by interacting with SAMHD1.

### NONO impairs DCAF1-mediated ubiquitination/degradation of SAMHD1

**Fig. 3:**
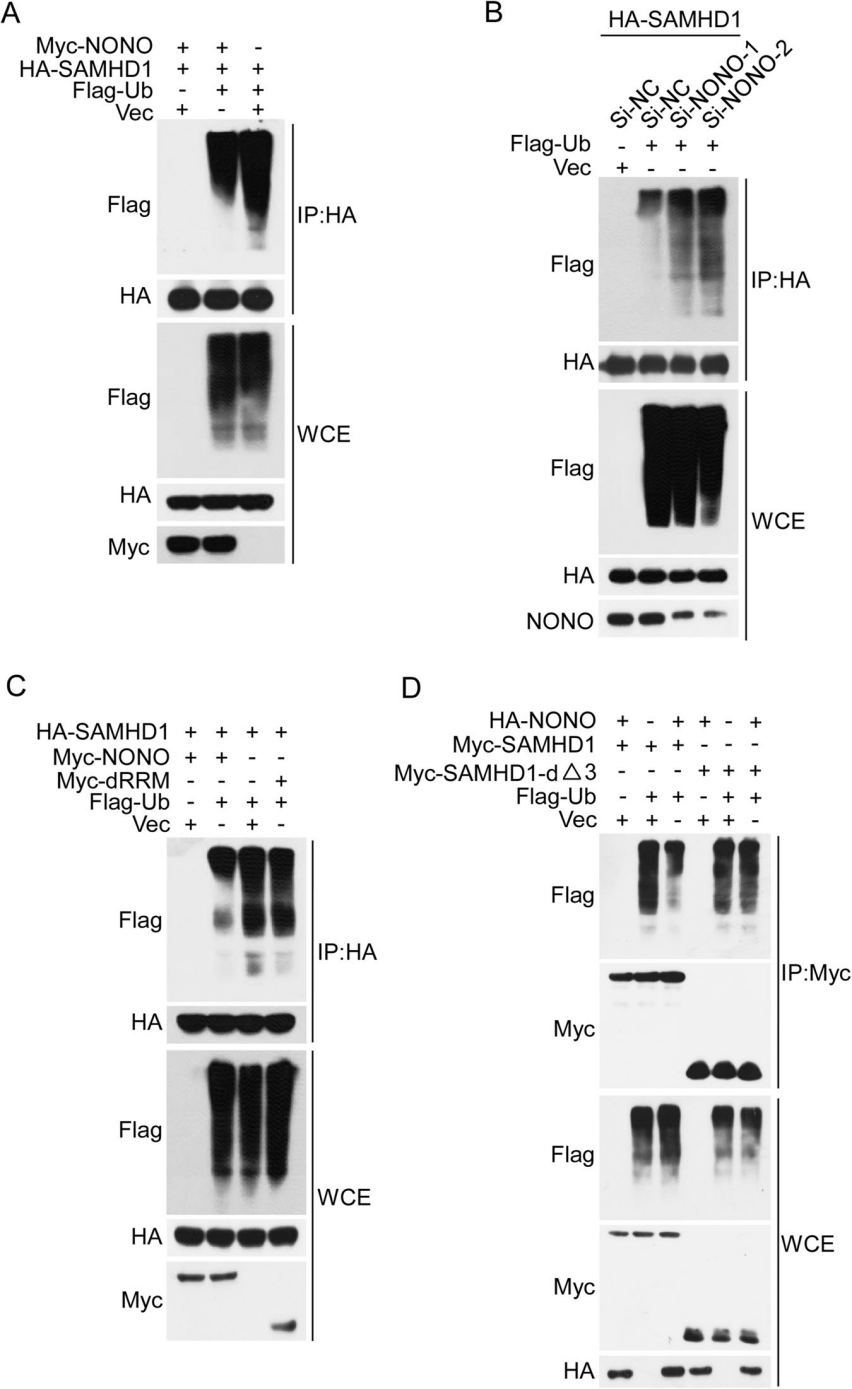
NONO inhibits the ubiquitination of SAMHD1. **A**, **B** Overexpression or silencing of NONO decreased or increased the ubiquitination level of SAMHD1, respectively. **A** HA-tagged SAMHD1 was cotransfected with Myc-tagged NONO and Flag-ubiquitin or empty vector into 293 T cells for 24 h. The SAMHD1 protein was enriched by IP, and the ubiquitination level of SAMHD1 was determined by western blotting. **B** 293 T cells transfected with NONO or negative control siRNAs for 24 h were then cotransfected with HA-tagged SAMHD1 and Flag-ubiquitin or empty vector for another 24 h, and the ubiquitination level of SAMHD1 was examined by IP followed by western blotting. **C** The NONO truncation mutant with deletion of the RRM domain did not affect the ubiquitination of SAMHD1. HA-tagged SAMHD1 was cotransfected with wild-type or mutant Myc-NONO and Flag-ubiquitin into 293 T cells for 24 h, and the ubiquitination level of SAMHD1 was examined by IP followed by western blotting. **D** Deletion of amino acids 115-562 in SAMHD1 abrogated the influence of NONO on SAMHD1 ubiquitination. Wild-type or mutant Myc-tagged SAMHD1 was cotransfected with HA-NONO and Flag-ubiquitin or empty vector into 293 T cells for 24 h, and the ubiquitination of SAMHD1 was evaluated using IP followed by western blotting.

To explore the underlying mechanisms by which NONO improves the stability of SAMHD1, we examined whether NONO influences the ubiquitination of SAMHD1. Overexpression of NONO decreased but the silencing of NONO increased the ubiquitination level of SAMHD1 (Fig. 3A, B). Consistent with its detrimental effect on the SAMHD1 protein level, deletion of the RRM domain of NONO abrogated the inhibitory effect of NONO on the ubiquitination of SAMHD1 (Fig. 3C). Similarly, NONO transfection did not affect the ubiquitination level of the SAMHD1 mutant with deletion of amino acids 115-562, the domain controlling its interaction with NONO (Fig. 3D). Taken together, these results indicated that NONO inhibits the ubiquitination of SAMHD1.

**Fig. 4:**
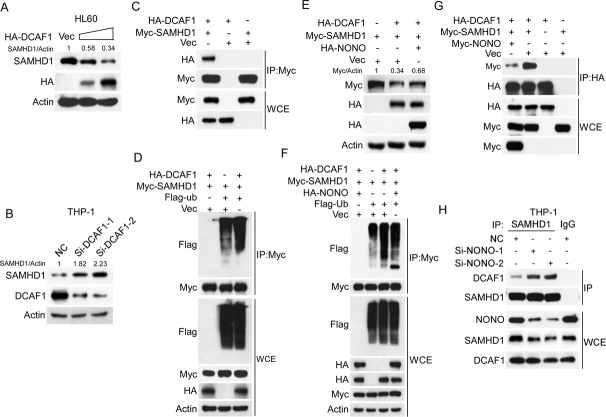
NONO blocks the ubiquitination-mediated degradation of SAMHD1 by DCAF1. **A**, **B** Overexpression or silencing of DCAF1 decreased or increased SAMHD1 expression, respectively. **A** HL60 cells were transfected with increasing concentrations of HA-tagged DCAF1 24 h, and the levels of the indicated proteins were determined by western blotting. **B** THP-1 cells were transfected with DCAF1 siRNAs for 24 h, and the levels of the indicated proteins were then determined by western blotting. **C** DCAF1 interacts with SAMHD1. 293 T cells were transfected with HA-tagged DCAF1 and Myc-tagged SAMHD1 for 24 h. Co-IP was performed with anti-Myc agarose, and the interactions were examined by western blotting. **D** Overexpression of DCAF1 increased the ubiquitination level of SAMHD1. Myc-tagged SAMHD1 was cotransfected with HA-tagged DCAF1, Flag-ubiquitin or empty vector into 293 T cells for 24 h, and the ubiquitination level of SAMHD1 was determined using IP followed by western blotting. **E** NONO blocked DCAF1-mediated degradation of SAMHD1. Myc-tagged SAMHD1 and HA-tagged DCAF1 were cotransfected into 293 T cells with or without overexpression of HA-tagged NONO for 24 h. The levels of the indicated proteins were determined by western blotting. **F** Overexpression of NONO inhibited DCAF1-mediated ubiquitination of SAMHD1. Myc-tagged SAMHD1, HA-tagged DCAF1, and Flag-tagged ubiquitin were cotransfected into 293 T cells with or without overexpression of HA-tagged NONO for 24 h. The ubiquitination of SAMHD1 was evaluated by IP followed by western blotting. **G** NONO inhibits the interaction of DCAF1 and SAMHD1. Myc-tagged SAMHD1 and HA-tagged DCAF1 were cotransfected into 293 T cells with or without overexpression of Myc-tagged NONO for 24 h. Cells were subsequently subjected to co-IP followed by western blotting with the indicated antibodies. **H** Silencing of NONO increases the interaction of DCAF1 with SAMHD1. THP-1 cells silencing NONO for 48 h were lysed and were subsequently subjected to co-IP followed by western blotting with the indicated antibodies. For respective immunoblots, the protein levels were quantified by ImageJ software.

Previous research has reported that the TRIM21 E3 ubiquitination ligase mediates the degradation of SAMHD1 to facilitate EV71 infection of rhabdomyosarcoma RD cells and 293 T cells [24]. We thus examined whether TRIM21 mediates the degradation of SAMHD1 in AML cells and found that both overexpression and silencing of TRIM21 had little effect on SAMHD1 expression in AML cells (Supplementary Fig. S3A, B). The DCAF1 E3 ligase has been reported to be hijacked by Vpx or Vpr to degrade SAMHD1 in immune cells upon viral infection [25]. Thus, we sought to determine whether DCAF1 mediates SAMHD1 degradation under physiological conditions in the absence of Vpx and Vpr. Indeed, ectopic expression of DCAF1 was reduced and silencing of DCAF1 increased the SAMDH1 protein level in AML cells (Fig. 4A, B). In addition, a co-IP assay result showed that DCAF1 interacted with SAMHD1 (Fig. 4C). Finally, overexpression of DCAF1 increased the ubiquitination of SAMHD1 (Fig. 4D). These results indicate that DCAF1 may be the crucial E3 ligase responsible for the degradation of SAMHD1 in AML cells. We then examined whether NONO affects the DCAF1-mediated degradation of SAMHD1. As shown in Fig. 4E, F, overexpression of NONO reversed the DCAF1-mediated downregulation of SAMHD1, restoring SAMHD1 expression to a level comparable to that in the negative control cells (Fig. 4E), reduced DCAF1-mediated ubiquitination of SAMDH1 (Fig. 4F), and decreased the binding of DCAF1 to SAMHD1 (Fig. 4G). We further examined whether the silence of NONO affected the interaction of DCAF1 and SAMHD1 in AML cells. The results showed that the silencing of NONO obviously increased the interaction between DCAF1 and SAMHD1 in THP-1 cells (Fig. 4H). Collectively, these results indicate that NONO improves the stability of SAMHD1 by blocking the DCAF1-mediated ubiquitination of SAMHD1.

### NONO mediates the sensitivity of AML cells to Ara-C

**Fig. 5:**
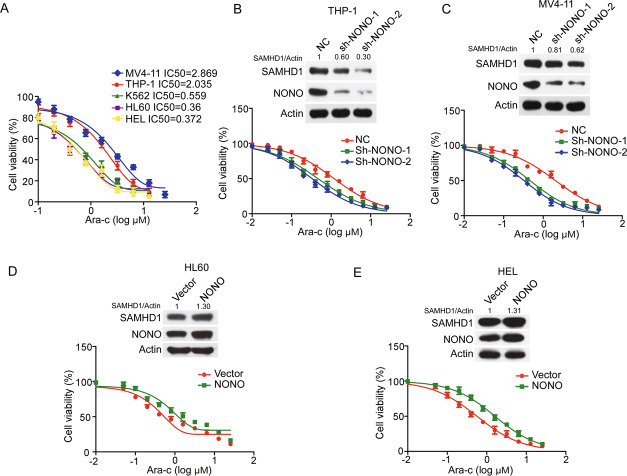
NONO affects the sensitivity of AML cells to Ara-C. **A** IC50 values of Ara-C in AML cell lines. AML cells (1 × 10^5^ cells/well) plated in 24-well plates were incubated for 24 h and were then treated with a series of concentrations of Ara-C for 48 h. Cell viability was evaluated by a CCK-8 assay (*n* = 3, mean ± SD). **B**, **C** Silencing NONO increased the sensitivity of AML cells to Ara-C. THP-1 and MV4-11 cells with stable knockdown of NONO were treated with different concentrations of Ara-C for 48 h, and cell viability was evaluated by a CCK-8 assay (*n* = 3, mean ± SD). **D**, **E** Overexpression of NONO decreased the sensitivity of AML cells to Ara-C. HL60 and HEL cell lines with stable overexpression of NONO were generated and treated with different concentrations of Ara-C for 48 h. Cell viability was evaluated by a CCK-8 assay (*n* = 3, mean ± SD). **B**–**E**, two-way ANOVA was used to compare the behavior of NONO-silenced or overexpressed and control cells. For respective immunoblots, the protein levels were quantified by ImageJ software.

Since SAMHD1 was found to be crucial for the sensitivity of AML cells to Ara-C, we sought to determine whether NONO regulates the sensitivity of AML cells to Ara-C. First, the half-maximal inhibitory concentration (IC50) values of Ara-C in AML cells were positively correlated with the NONO protein levels (Fig. 2E and Fig. 5A). In addition, silencing of NONO increased the sensitivity of THP-1 and MV4-11 cells to Ara-C, as evaluated by proliferation and apoptosis assays (Fig. 5B, C and Supplementary Fig. S4A, B). In contrast, overexpression of NONO in HL60 and HEL cells reduced their sensitivity to Ara-C (Fig. 5D, E). These results indicate that NONO is implicated in the sensitivity of AML cells to Ara-C.

**Fig. 6:**
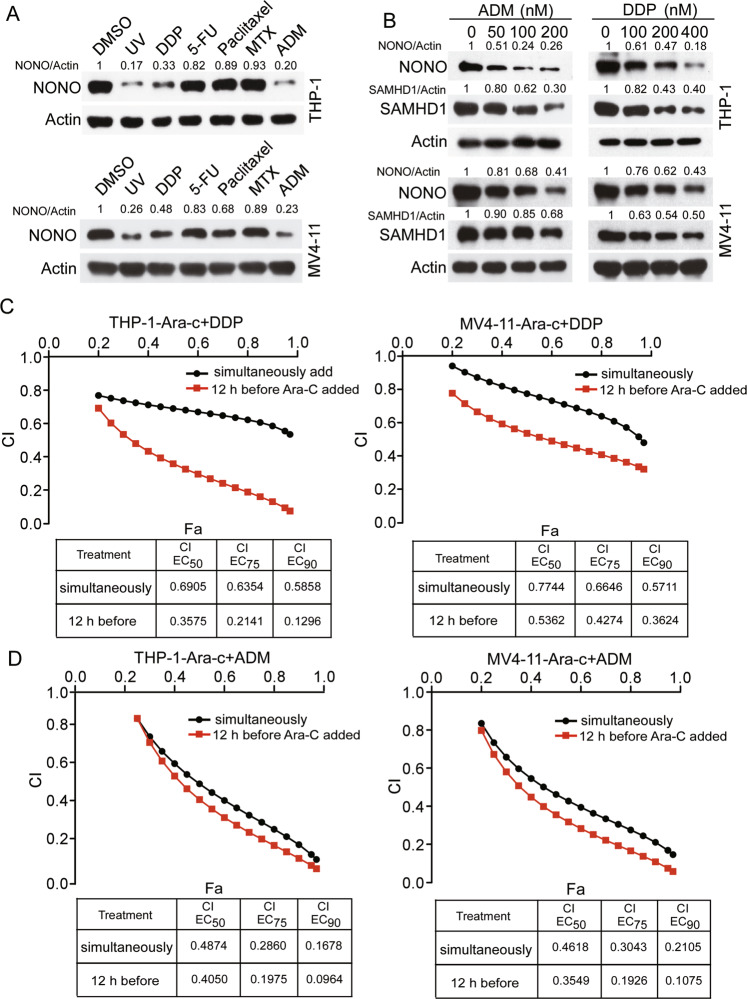
DDP and ADM show synergistic effects with Ara-C in killing AML cells. **A** Screening of the potential chemical agents able to downregulate NONO expression. THP-1 and MV4-11 cells were treated with the indicated concentrations of drugs, including DDP (1 μM), 5-FU (2 μM), paclitaxel (3.5 μM), MTX (0.1 μM), and ADM (0.1 μM), for 48 h or with UV radiation (30 J/m^2^), and the levels of the indicated proteins were evaluated by western blotting. DMSO was used as the negative control. **B** DDP and ADM downregulated the protein expression of NONO and SAMHD1 in AML cells. THP-1 and MV4-11 cells were treated with the indicated concentrations of drugs for 48 h, and the levels of the indicated proteins were evaluated by western blotting. **C**, **D** DDP and ADM sensitized AML cells to Ara-C. Plots of the CIs for Ara-C and DDP (**C**) or ADM (**D**) in THP-1 and MV4-11 cells, as determined using the Chou–Talalay method (*n* = 3). Red lines: AML cells were treated with DDP or ADM for 12 h and then added with a series concentration of Ara-C for 36 h. Black lines: Ara-C and DDP or ADM were added simultaneously into AML cells and then incubated for 48 h. For respective immunoblots, the protein levels were quantified by ImageJ software.

NONO has been reported to be degraded upon UV-mediated DNA single-strand breaks or IR-mediated DNA double-strand breaks [31]. We sought to explore the potential of DNA damage-related chemical agents to induce NONO degradation and sensitize AML cells to Ara-C. We screened a series of chemotherapeutic agents and, as expected, found that like UV irradiation, DDP and ADM treatment obviously reduced NONO expression (Fig. 6A) and the level of its downstream protein SAMHD1 in a dose-dependent manner in both THP-1 and MV4-11 cells (Fig. 6B). In addition, drug combination assays showed that both DDP and ADM exhibited synergistic effects with Ara-C in killing THP-1 and MV4-11 cells (Fig. 6C, D). Notably, treatment with DDP or ADM 12 h before the addition of Ara-C had stronger synergistic effects in AML cells than simultaneous addition of the two chemical agents (Fig. 6C, D), indicating that this synergism is dependent on the downregulation of NONO/SAMHD1 before Ara-C treatment.

### NONO is crucial for the resistance of AML cells to Ara-C

**Fig. 7:**
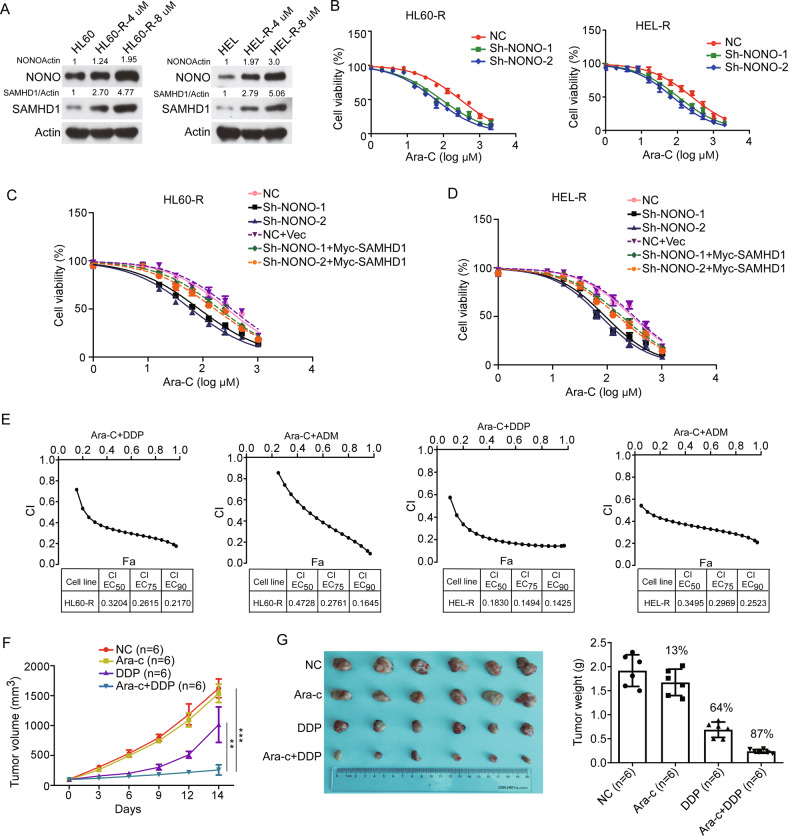
NONO is crucial for the resistance of AML cells to Ara-C. **A** Both NONO and SAMHD1 were upregulated in AML-resistant cells compared with the corresponding parental cells. The protein levels of NONO and SAMHD1 in HL60-R and HEL-R cells and the corresponding parental cells were evaluated by western blotting (P indicates parental cells. HL60-R-4 μM and HL60-R-8 μM, HEL-R-4 μM, and HEL-R-8 μM indicate the maximum sustained concentrations of Ara-C used for culture of the resistant AML cell lines). **B** Silencing NONO restored the sensitivity of resistant AML cells to Ara-C. Resistant AML cells with or without stable NONO silencing were treated with Ara-C at a series of concentrations as indicated for 48 h, and cell viability was evaluated by a CCK-8 assay (*n* = 3, mean ± SD). **C**, **D** HL60-R (**C**) and HEL-R (**D**) cells with or without stably silencing NONO were transfected with SAMHD1 or Vector for 24 h. Then the cells were treated with Ara-C at the indicated concentrations for 48 h, and cell viability was evaluated by CCK-8 assay (*n* = 3, mean ± SD). **E** DDP and ADM sensitized resistant AML cells to Ara-C. Plots of the CIs for Ara-C and DDP or ADM in HL60-R and HEL-R cells, as determined using the Chou–Talalay method (*n* = 3). **F** NOD/SCID mice bearing HL60-R cell xenografts were treated with PBS, Ara-C, DDP, or a combination of Ara-C and DDP, and tumor growth was monitored. *n* = 6 for each group. **G** The tumor weights for each group as indicated in Fig. 7F. In B-D, 2-way ANOVA was used to compare the behavior of the cells treated as indicated and negative control cells. **F**, **G**, a two-tailed unpaired Student’s *t* test was used to determine statistical significance (**P < 0.01, ***P < 0.001). For respective immunoblots, the protein levels were quantified by ImageJ software.

To explore whether NONO might contribute to the resistance of AML cells to Ara-C, we gradually adapted HL60 and HEL cells, characterized by low NONO protein levels and high sensitivity to Ara-C, to grow in the presence of Ara-C. The resulting resistant HL60 and HEL cell lines (designated HL60-R and HEL-R, respectively) were cultured in a medium containing Ara-C at a maximum concentration of 8 μM, and the IC50 values of Ara-C in these cells were 1000-fold greater than those in the corresponding parental cell lines (Supplementary Fig. S5A). Moreover, the protein levels but not mRNA levels of SAMHD1 were obviously increased in the resistant AML cell lines compared to the corresponding parental cell lines (Fig. 7A and Supplementary Fig. S5B). Consistently, the protein levels of NONO were upregulated in resistant AML cells, indicating that NONO may be responsible for the increased SAMHD1 protein level and subsequent Ara-C resistance in resistant AML cells. We then stably knocked down NONO in HL60-R and HEL-R cells (Supplementary Fig. S6A) and found that silencing NONO partially restored the sensitivity of these two resistant AML cell lines to Ara-C, as evaluated by proliferation and apoptosis assays (Fig. 7B and Supplementary Fig. S6B, C). Consistently, the Introduction of SAMHD1 into the NONO-silenced HL60-R and HEL-R cells restored at least in part their resistance to Ara-C (Fig. 7C, D). Similarly, treatment with either DDP or ADM reversed the resistance of resistant AML cells to Ara-C, as the calculated CI for the combination of Ara-C with DDP ranged from 0.9 to 0.2 in HL60-R cells and HEL-R cells, and the CI for the combination of Ara-C with ADM ranged from 0.9 to 0.1 in HL60-R cells and from 0.5 to 0.2 in HEL-R cells (Fig. 7E). More importantly, resensitization of resistant AML cells to Ara-C by DDP was also observed in the AML xenograft model. The tumor growth and weight of xenografts tumors from HL60-R cells were inhibited by DDP alone and even more efficiently reduced by the combination of Ara-C with DDP but were slightly affected by Ara-C alone (Fig. 7F, G).

## Discussion

**Fig. 8:**
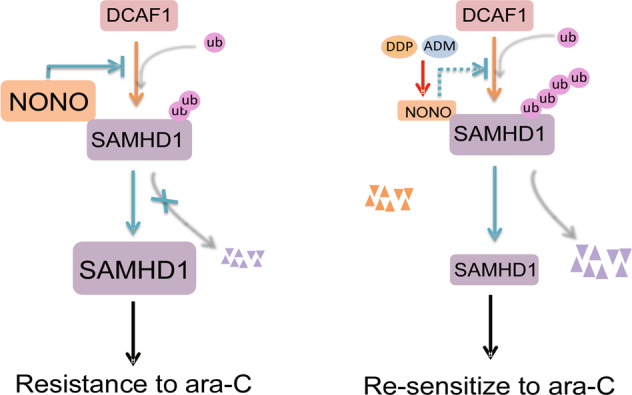
Schematic representation of the molecular mechanism of NONO stabilizing SAMHD1 and the functional role of targeting NONO using ADM and DDP to resensitize resistant AML cells to Ara-C.

In this study, we revealed that NONO interacts with and stabilizes SAMHD1 by inhibiting its ubiquitination/degradation mediated by the DDB1-DCAF1 E3 ligase. Upregulation of NONO expression leads to upregulation of SAMHD1 in AML cells, which leads to the resistance of AML cells to Ara-C. Downregulation of NONO by siRNAs increases the sensitivity of AML cells to Ara-C. Importantly, we found that DNA damage reagents ADM and DDP could downregulate NONO expression, which increases the cytotoxicity of Ara-C to AML cells and resensitize resistant AML cells to Ara-C (Fig. 8).

NONO is a multifunctional protein implicated in multiple physiological and pathological processes, including DNA damage repair, metabolism, cancer progression, and chemoresistance [32–38]. NONO is involved in almost every aspect of gene regulation, including mRNA splicing, DNA unwinding, transcriptional regulation, nuclear retention of defective RNA, and DNA repair [39–43]. In addition, we and other research groups found that NONO can stabilize its interacting partners [30, 44]. In this study, we found that NONO interacts with and stabilizes SAMHD1. Overexpression of NONO obviously prolonged the half-life of SAMHD1, whereas silencing of NONO shortened its half-life. Two E3 ligases have been reported to mediate the ubiquitination/degradation of SAMHD1, namely, TRIM21, which promotes the proteasomal degradation of SAMHD1 upon EV71 infection, and DDB1-DCAF1, which is hijacked by the viral Vpx or Vpr protein to promote SAMHD1 degradation during simian immunodeficiency virus (SIV) infection [24, 27]. In our study, we found that silencing TRIM21 in AML cells had little effect on SAMHD1 expression, indicating that the ubiquitination-mediated degradation of SAMHD1 by TRIM21 is context-dependent. We then examined whether DCAF1 can ubiquitinate and degrade SAMHD1 in AML cells without the assistance of Vpx or Vpr. We found that DCAF1 interacted with both endogenous and exogenous SAMHD1. In addition, silencing or overexpression of DCAF1 increased or reduced SAMHD1 expression, respectively, in AML cells. These results indicate that Vpx is dispensable for the DCAF1-mediated turnover of SAMHD1 in AML cells. We speculated that the stabilization of SAMHD1 by NONO is mediated through the blockade of ubiquitination/degradation by DCAF1. Indeed, overexpression of NONO attenuated both the interaction of SAMHD1 with DCAF1 and its ubiquitination by DCAF1.

The SAMHD1 expression level has been reported to be correlated with the sensitivity of AML cells to Ara-C [9]. Rudd et al. reported that the ribonucleotide reductase inhibitors gemcitabine and hydroxyurea suppress the Ara-CTPase activity of SAMHD1 and overcome the SAMHD1-mediated barrier to Ara-C efficacy in both primary blasts and mouse models of AML [45]. In addition, Vpx-mediated downregulation of SAMHD1 sensitizes AML cells to Ara-C [10]. These results suggest that suppression or downregulation of SAMHD1 would sensitize AML cells to Ara-C. However, no clinically validated SAMHD1-specific inhibitors have been developed. Since NONO improved the stability of SAMHD1, we speculated that NONO would affect the sensitivity of AML cells to Ara-C. Using a cell viability assay, we found that silencing NONO obviously increased the sensitivity of AML cells to Ara-C. We then explored potential chemical agents that could downregulate NONO expression in AML cells. UV radiation-mediated single-stranded DNA break and IR radiation-mediated double-stranded DNA break have been reported to reduce the NONO protein level [31, 46, 47]. We thus examined whether chemical DNA-damaging agents can lead to NONO turnover. Our findings showed that in addition to UV radiation, treatment with either DDP or ADM obviously reduced NONO expression. This result prompted us to explore whether DDP and ADM might sensitize AML cells to Ara-C. Our results confirmed that both DDP and ADM showed synergistic effects with Ara-C in AML cells. More importantly, combination Ara-C has been used in combination with DDP or ADM in the clinical treatment of AML patients for more than three decades, and their synergistic effect in AML patients has been clinically validated [48, 49].

Acquired resistance to Ara-C has made the clinical application of this drug and patient care challenging [50]. Several studies have reported that SAMHD1, whose expression is upregulated in Ara-C-resistant AML patients, is one of the most important factors promoting Ara-C resistance [9, 18]. However, the mechanism by which SAMHD1 is upregulated in resistant AML cells is unknown. Here, we found that NONO expression is elevated in resistant AML cells, leading to SAMHD1 stabilization, which may contribute to the upregulation of SAMHD1 in resistant AML cells. In addition, as shown in Fig. 7C-E, both DDP and ADM resensitized AML cells to Ara-C both in vitro and in the xenograft model.

Overall, our findings revealed a potential mechanism by which DDP and ADM show clinically synergistic effects with Ara-C in AML patients and suggest a strategy to overcome Ara-C resistance in AML.

## Supplementary information

Supplementary file

reproducibility-checklist

## Data Availability

All data generated or analyzed during this study are included in this published article [and its supplementary information files].
