# Supplementary material for: Stabilization of SAMHD1 by NONO is crucial for Ara-C resistance in AML
Source: Cell Death Dis. 2022 Jul 8;13(7):590. doi: 10.1038/s41419-022-05023-0 (PMC9270467; doi:10.1038/s41419-022-05023-0)
Supplement: Supplementary file 1 — Supplementary file [file 41419_2022_5023_MOESM1_ESM.docx]

**Supplementary Figures and Supplementary Table S for**

**Stabilization of SAMHD1 by NONO is crucial for Ara-C resistance in AML**

Feifei Zhang^1, *^, Jun Sun^1,2, *^, Xiaofeng Tang^1, *^, Yiping Liang^1^, Quanhui Jiao^1,2^, Bo Yu^1,3^, Zhengzai Dai^1,3^, Xuhui Yuan^1,3^, Jiayu Li^1,3^, Jinhua Yan^1^, Zhiping Zhang^1,3^, Song Fan^4^, Min Wang^5^, Haiyan Hu^6, #^, Changhua Zhang^2, #^, Xiao-Bin Lv^1, #^

* These authors contributed equally to this work.

# **Address correspondence Author to:**

Xiao-Bin Lv: Jiangxi Key Laboratory of Cancer Metastasis and Precision Treatment, Central Laboratory, The First Hospital of Nanchang, The Third Affiliated Hospital of Nanchang University, North 128 Xiangshan Road, Nanchang, 330008, China. Phone: +86-791-88862438; Emial: nclvxiaobin@sina.cn.

Changhua Zhang: College of Pharmacy, Jiangxi University of Chinese Medicine, Nanchang, 330004, China. Emial: zhangch305@126.com.

Haiyan Hu: Oncology Department of Shanghai Jiao Tong University Affiliated Sixth People's Hospital, Shanghai, 200233, China. Emial: xuri1104@163.com.


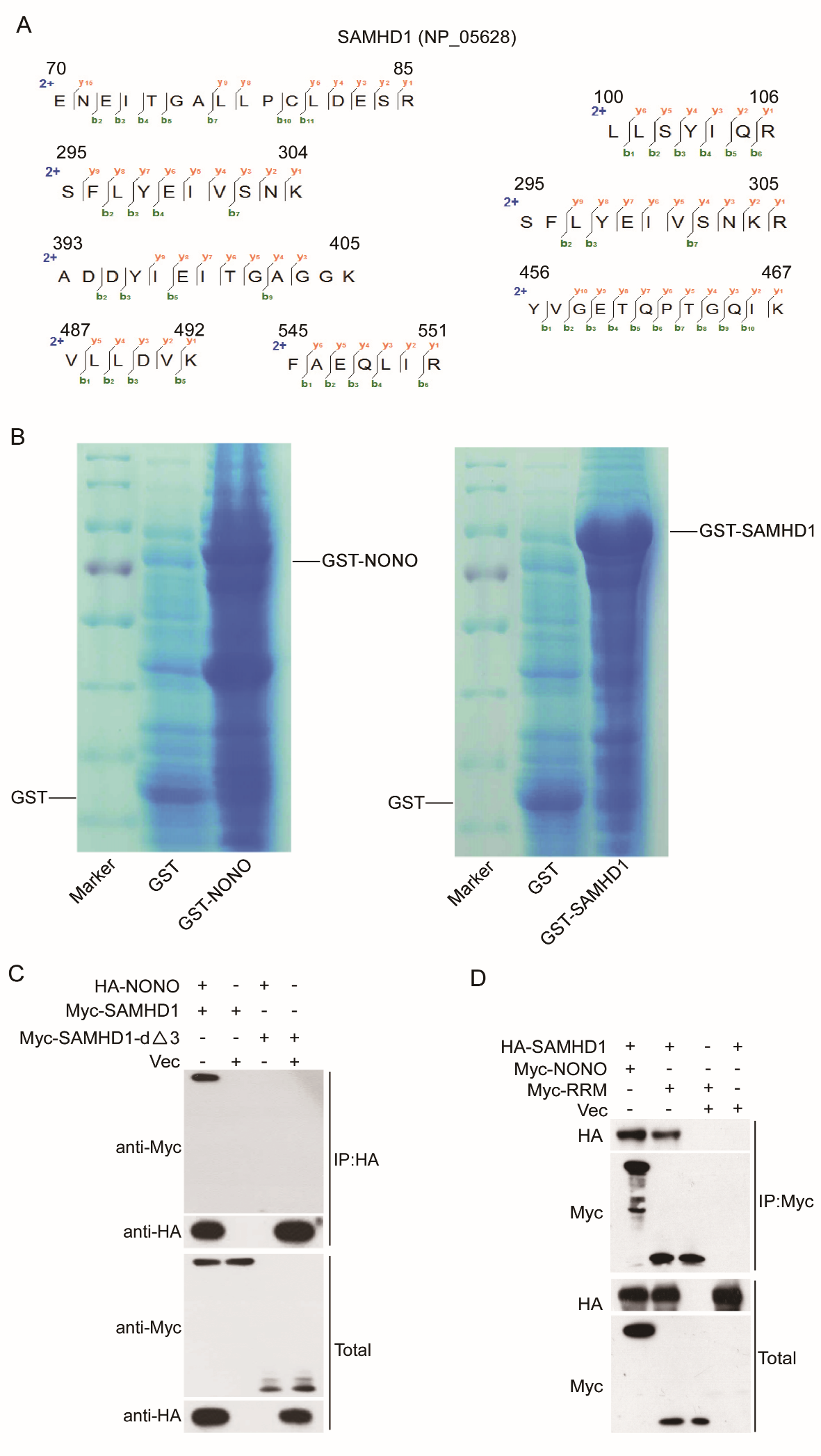


**Supplementary Fig. S1.** **Secondary mass spectrum of SAMHD1 and NONO interacts with SAMHD1.** **(A)** Secondary mass spectrum showing the peptides from SAMHD1 present in the NONO protein complex. **(B)** GST-NONO (left) and GST-SAMHD1 (right) proteins expressed in an BL21 E. coli were detected by SDS-PAGE with Coomassie staining. BL21 E. coli cells transformed with GST-NONO, GST-SAMHD1 or GST were supplied with 50 ug/mL of IPTG and incubated for 6 hours. The whole lysates were separated by SDS-PAGE with Coomassie staining and GST-NONO, GST-SAMHD1 or GST proteins were indicated as arrows. **(C)** Deletion of amino acids 115-562 in SAMHD1 abrogates its interaction with NONO. Wild-type or truncated SAMHD1 was cotransfected with HA-tagged NONO into 293T cells, and their interactions were evaluated by co-IP and western blotting. **(D)** The RRM domain of NONO is sufficient for the interaction of NONO with SAMHD1. Wild-type or mutant Myc-NONO was cotransfected with HA-tagged SAMHD1 into 293T cells, and the interactions were evaluated by co-IP and western blotting.





**Supplementary Fig. S2.** **SAMHD1 did not affect the protein level of NONO and NONO did not affect the mRNA level of SAMHD1.** **(A)** Overexpression of SAMHD1 did not affect the protein level of NONO. 293T cells were cotransfected with Myc-tagged NONO and increasing concentrations of HA-tagged SAMHD1 for 24 hours, and the levels of the indicated proteins were evaluated by western blotting. **(B)** Silencing SAMHD1 did not affect the protein level of NONO. 293T cells transfected with SAMHD1 or negative control siRNAs for 24 hours were then transfected with Myc-tagged NONO for 24 hours. The levels of the indicated proteins were evaluated by western blotting. **(C-D)** Silencing or overexpressing NONO did not affect the mRNA level of SAMHD1. HL60 cells were transfected with NONO siRNAs for 72 hours **(C)**, and HL60 cells were transfected with the NONO expression vector for 24 hours **(D)**. The mRNA level of SAMHD1 was determined by qRT–PCR (n=3, mean ± SD). Two-tailed unpaired Student’s t test was used to determine statistical significance (** p<0.01; n.s., not significant). For respective immunoblots, the protein levels were quantified by ImageJ software.





**Supplementary Fig. S3.** **TRIM21 did not affect the protein level of SAMHD1.** **(A)** Overexpression of TRIM21 did not affect the protein level of SAMHD1. HL-60 cells were transfected with increasing concentrations of Myc-tagged TRIM21 for 24 hours, and the levels of the indicated proteins were evaluated by western blotting. **(B)** Silencing TRIM21 did not affect the protein level of SAMHD1. THP1 cells were transfected with TRIM21 or negative control siRNAs for 72 hours, and the levels of the indicated proteins were determined by western blotting. For respective immunoblots, the protein levels were quantified by ImageJ software.


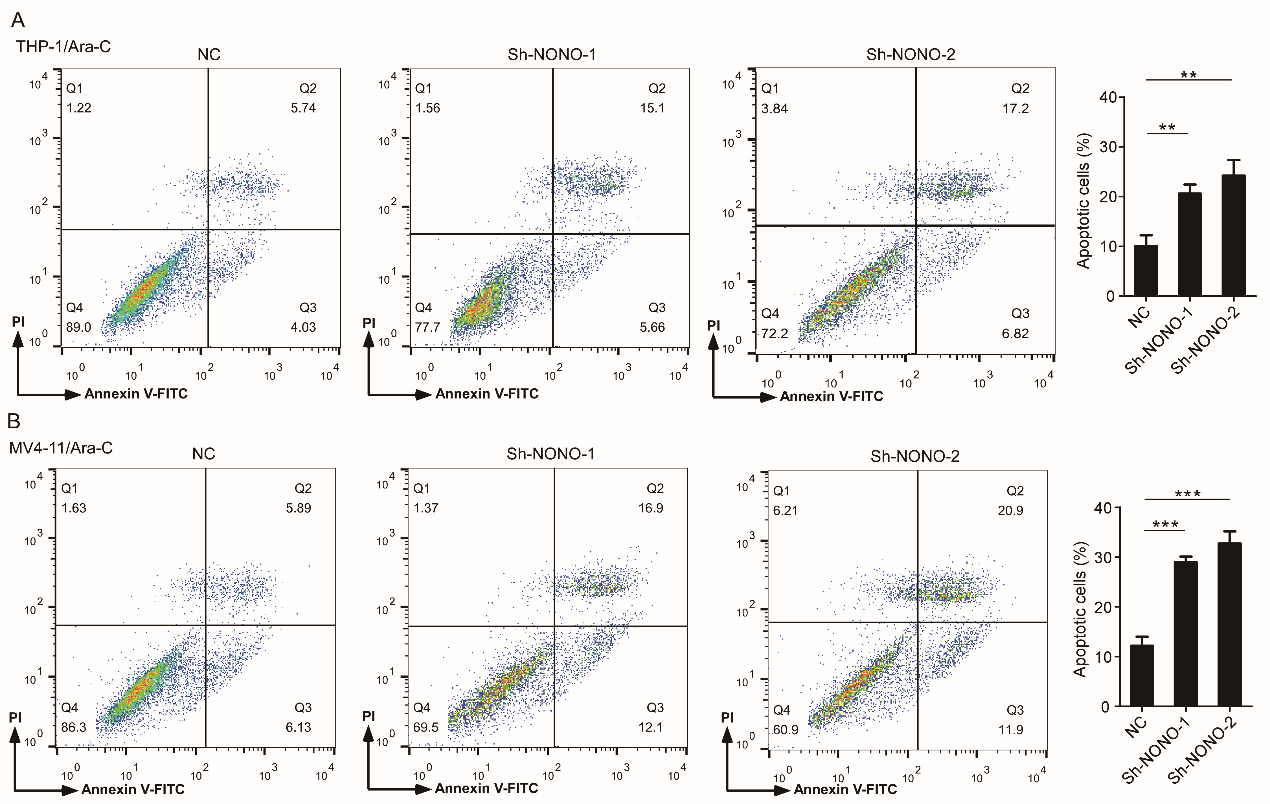


**Supplementary Fig. S4. NONO knockdown improves Ara-C induced cellular apoptosis in AML cells.** **(A)** THP-1 cells with or without silencing NONO were treated with 0.8 µM of Ara-C for 48 hours and the cellular apoptosis was assessed by flow cytometry (left). The quantified data were also shown (right). **(B)** MV4-11 cells with or without silencing NONO were treated with 1.6 µM of Ara-C for 48 hours and the cellular apoptosis was assessed by flow cytometry (left). The quantified data were also shown (right). (n=3, mean ± SD), two-tailed unpaired Student’s t test was used to determined statistical significance (** p<0.01, *** p<0.001).


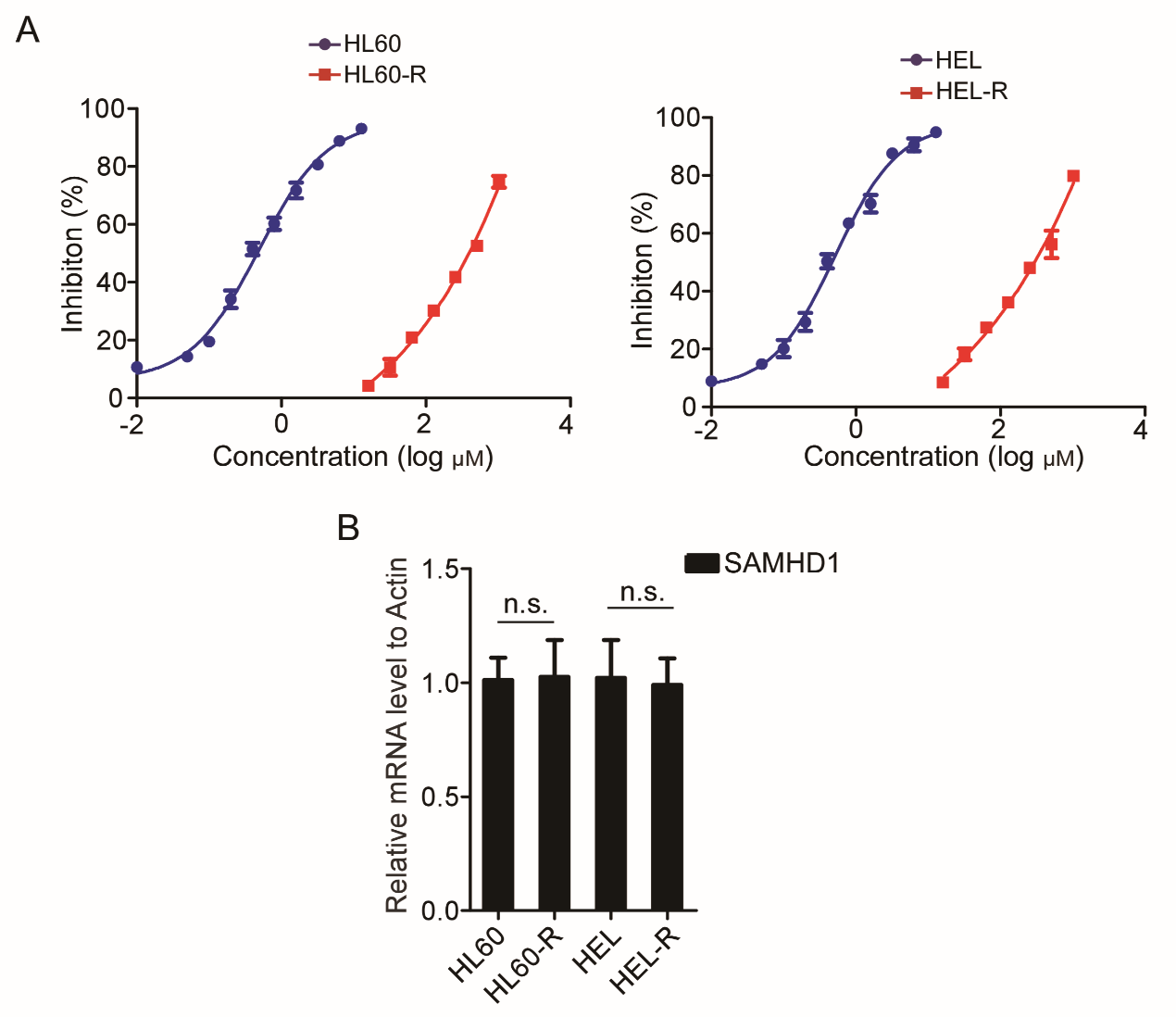


**Supplementary Fig. S5.** **The IC50 values of Ara-C and mRNA level of SAMHD1 in the parental and resistant AML cells.** **(A)** The IC50 values of Ara-C in the indicated resistant AML cells. AML-resistant cells were treated with the indicated concentrations of Ara-C for 48 hours, and cell viability was then evaluated by a CCK-8 assay (n=3, mean ± SD). **(B)** The mRNA level of SAMHD1 in resistant melanoma cells and the corresponding parental cells as indicated was evaluated using qRT–PCR (n=3, mean ± SD). Two-tailed unpaired Student’s t test was used to determine statistical significance (n.s., not significant).

**
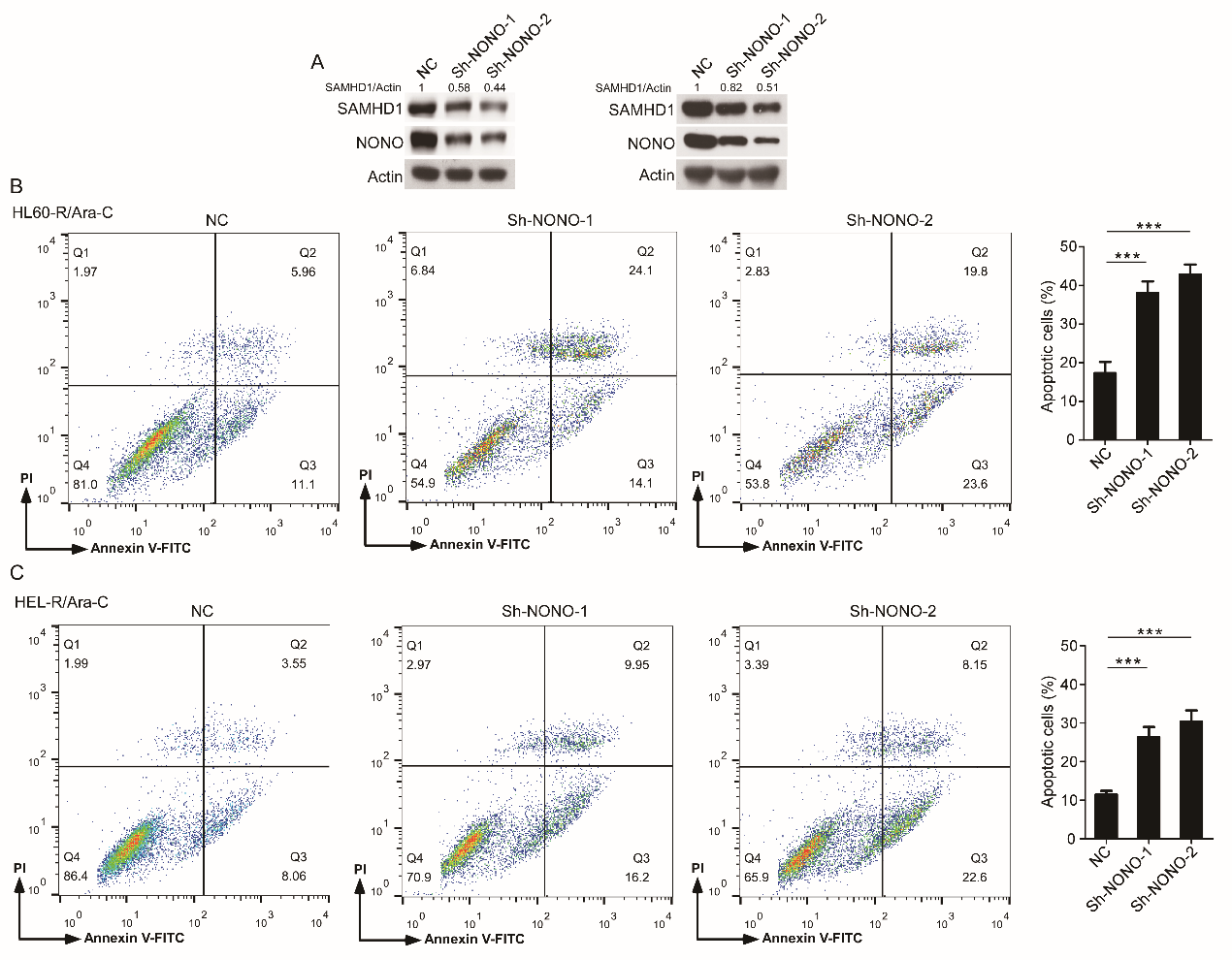
**

**Supplementary Fig. S6. NONO knockdown improves Ara-C induced cellular apoptosis in resistant AML cells.** **(A)** NONO and SAMHD1 protein levels in HL60-R (left), HEL-R (right) cells with or without stable knockdown were determined by western blotting. **(B-C)** HL60-R (B) and HEL-R (C) cells with or without silencing NONO were with 300 µM Ara-C and the cellular apoptosis was assessed by flow cytometry (left). The quantified data were also shown (right). (n=3, mean ± SD), two-tailed unpaired Student’s t test was used to determined statistical significance (*** p<0.001). For respective immunoblots, the protein levels were quantified by ImageJ software.

**Supplementary Table S. Information for primers, plasmids and siRNA sequences.**

| **Gene** | **Forward primer (5’-3’)** | | | **Reverse primer (5’-3’)** |
| --- | --- | --- | --- | --- |
| **qRT-PCR** | | | | |
| NONO | GGCAGGCGAAGTCTTCATTCA | | | TGGCAATCTCCGCTAGGGT |
| SAMHD1 | GGTCCGGAGCAGGTGTG | | | CAAAACGAGACTCATCAAGAC |
| β-Actin | CTCCTTAATGTCACGCACGAT | | | CATGTACGTTGCTATCCAGGC |
| **pSSH vector construction** | | | | |
| Streptavidin binding peptide sequence | | ATGGACGAGAAGACCACCGGCTGGAGAGGCGGCCACGTGGTGGAGGGCCTGGCCGGCGAGCTGGAGCAGCTGAGAGCCAGACTGGAGCACCACCCTCAGGGCCAGAGAGAACCT | | |
| S-protein binding peptide sequence | | AAAGAAACCGCTGCTGCTAAATTCGAACGCCAGCACATGGACAGC | | |
| HA-tags | | TACCCATACGATGTTCCAGATTACGCT | | |
| **Plasmids construction** | | | | |
| pcDNA3.1-HA-S-Streptavidin | | | AAGGATCCCAGAGTAATAAAACTTTTAACTTGGAG | TTCTCGAGTTAGTATCGGCGACGTTTGT |
| pLKO.1-  Sh-NONO-1 | | | CCGGGGCTTGACTATTGACCTGACTCGAGTCAGGTCAATAGTCAAGCCTTTTTTGGTACC | AATTGGTACCAAAAAAGGCTTGACTATTGACCTGACTCGAGTCAGGTCAATAGTCAAGCC |
| pLKO.1-  Sh-NONO-2 | | | CCGGCCAGCAATTTCACAAGGAACTCGAGTTCCTTGTGAAATTGCTGGTTTTTTGGTACC | AATTGGTACCAAAAAACCAGCAATTTCACAAGGAACTCGAGTTCCTTGTGAAATTGCTGG |
| pSSH-SAMHD1 | | | AATGGATCCCAGCGAGCCGATTCCGA | ACTCTCGAGTCACATTGGGTCATCTTTAAAAAGC |
| pSSH-TRIM21 | | | AATGATATCGCTTCAGCAGCACGCTTGA | ACTCTCGAGTCAATAGTCAGTGGATCCTTGTGAT |
| pSSH-DCAF1 | | | AATGATATCACTACAGTAGTGGTACATGTGGACTC | ACTCTCGAGTCACTCATTCAGAGATAAGATGATGT |
| **siRNA targeting sequences** | | | | |
| Si-NONO-1 | | GGCTTGACTATTGACCTGA | | |
| Si-NONO-2 | | CCAGCAATTTCACAAGGAA | | |
| Si-TRIM21-1 | | CAGCACGCUUGACAAUGAU | | |
| Si-TRIM21-2 | | GACUUCACCUGUUCUGUGA | | |
| Si-DCAF1-1 | | UCACAGAGUAUCUUAGAGA | | |
| Si-DCAF1-2 | | GCGACUCAUUCUCCAAUAU | | |
| Si-SAMHD1-1 | | GACAAUGAGUUGCGUAUUU | | |
| Si-SAMHD1-2 | | AAGUAUUGCUAGACGUGAA | | |
